# Enhanced Humoral and Cellular Immune Responses Elicited by a Liposomal Subunit Vaccine Based on *Gyrovirus homsa1* VP1 Protein in Chickens

**DOI:** 10.3390/vetsci13080801

**Published:** 2026-08-13

**Authors:** Mengshi Chen, Rongchang Liu, Xin Yang, Chuchu Duan, Xiaozhen Yu, Liyun Zhuang, Tingting Dai, Haiyu Chen, Zehua Jin, Zuchen Song, Xintian Zheng

**Affiliations:** 1 Fujian Provincial Key Laboratory of Preventive Veterinary Medicine and Veterinary Biotechnology, College of Life Sciences, Longyan University, Longyan 364000, China; 2 Fujian Provincial Key Laboratory for Avian Diseases Control and Prevention, Institute of Animal Husbandry and Veterinary Medicine, Fujian Academy of Agricultural Sciences, Fuzhou 350000, China; 3 College of Animal Sciences, Fujian Agriculture and Forestry University, Fuzhou 350000, China

**Keywords:** *Gyrovirus homsa1*, VP1 protein, liposomal nanoparticle, subunit vaccine, immunogenicity

## Abstract

*Gyrovirus homsa1* (GyH1) causes immunosuppression and multi-organ damage in young poultry. The VP1 capsid protein (VP1) is a key target for subunit vaccine development; however, recombinant VP1 alone elicits limited immunogenicity and requires an efficient delivery system. Here, we developed a liposomal formulation associated with the GyH1 VP1 protein (LNP-VP1) and evaluated its immunogenicity and preliminary safety in specific-pathogen-free chickens. The prepared LNP-VP1 had a mean particle size of 230.11 ± 5.95 nm, a polydispersity index of 0.204 ± 0.023, a zeta potential of −36.55 ± 0.99 mV, and an apparent encapsulation efficiency of 84.03% ± 2.10%. In addition, immunization of chicks with LNP-VP1 induced robust antigen-specific IgG responses and significantly enhanced the infiltration of CD4^+^ and CD8^+^ T cells in the spleen. Furthermore, cytokine analysis revealed upregulation of IFN-γ, IL-4, and IL-17 levels following vaccination. Importantly, no vaccine-associated histopathological changes were observed in major immune or metabolic organs after immunization. Overall, LNP-VP1 effectively elicited both humoral and cellular immune responses with a favorable safety profile, indicating its potential as a candidate vaccine against GyH1 and providing experimental evidence supporting lipid nanoparticles as a delivery platform for avian subunit vaccines.

## Simple Summary

*Gyrovirus homsa1* (GyH1) is an emerging virus associated with immunosuppression, growth retardation, and organ damage in young chickens. At present, no commercial vaccine is available specifically for GyH1. In this study, we prepared a liposomal formulation containing the major capsid protein VP1 and evaluated its ability to induce immune responses in specific-pathogen-free chickens. The formulation showed stable physicochemical properties and sustained antigen release under laboratory conditions. Chickens immunized with LNP-VP1 developed increased VP1-specific antibody responses, enhanced splenic CD4^+^ and CD8^+^ immunofluorescence signals, and elevated serum levels of IFN-γ, IL-4, and IL-17. No obvious adverse effects on growth, immune organ indices, or tissue morphology were observed under the experimental conditions. These findings indicate that the LNP-VP1 formulation is immunogenic and shows a favorable preliminary safety profile in chickens. However, virus neutralization and GyH1 challenge experiments were not performed; therefore, its protective efficacy against infection remains to be determined.

## 1. Introduction

*Gyrovirus homsa1* (GyH1) is a newly identified gyrovirus (family *Anelloviridae*) that has been widely detected in chicken flocks and wild birds in China in recent years [1,2]. Infection with GyH1 leads to growth retardation, aplastic anemia, severe immunosuppression, and multi-organ damage in young poultry, posing a persistent economic and biosecurity threat to the poultry industry [1,3]. Serological surveillance has revealed a high prevalence of GyH1 infection across multiple provinces in China, with younger birds showing a significantly higher risk [4], suggesting that young chicks may be a key target for future control strategies. However, apart from the closely related chicken anemia virus (CAV), no commercial vaccine is currently available against viruses of the genus Gyrovirus, leaving GyH1 infection without specific intervention. Therefore, the development of safe and effective vaccines against GyH1 has become an urgent priority [1]. As the major capsid protein of GyH1, VP1 plays a critical role in viral assembly and infection and has been demonstrated to possess favorable immunogenicity and high conservation [2,3,4]. Consequently, the development of a subunit vaccine based on recombinant VP1 protein is well grounded in theory and offers distinct targeting advantages.

However, protein subunit vaccines generally suffer from weak immunogenicity and thus require efficient delivery systems and adjuvants to elicit robust protective immune responses. Liposomes and their derivatives, lipid nanoparticles (LNPs), have emerged as well-established vaccine delivery platforms capable of efficiently encapsulating and protecting protein antigens. Their uniform nanoscale size and surface properties facilitate uptake by antigen-presenting cells and drainage to lymphoid tissues, thereby enhancing antigen immunogenicity [5,6]. Moreover, LNPs possess intrinsic adjuvant activity; they promote antigen retention and sustained release at the injection site, enhance the activation of antigen-presenting cells, and induce durable and balanced humoral and cellular immune responses [7,8]. The potential application of LNPs in avian vaccines has been preliminarily explored in several studies [9,10]. Subcutaneous injection, a commonly used immunization route in poultry, offers ease of operation, standardization, and the ability to induce stable systemic immune responses, making it well suited for vaccination protocols in large-scale farming [11].

Thus, the development of an LNP-based subunit vaccine encapsulating the GyH1 VP1 protein (LNP-VP1) holds significant scientific value and application potential. Nevertheless, this strategy remains completely unexplored for GyH1 control, and the optimal formulation, immune response profile, and safety of such a vaccine have yet to be elucidated. In this study, we constructed an LNP-VP1 vaccine, systematically characterized its physicochemical properties and in vitro release behavior, and comprehensively evaluated its immunogenicity, including both humoral and cellular immune responses, as well as its safety in an SPF chicken model following subcutaneous administration. Our findings aim to provide experimental evidence for the development of a novel, efficient, and safe vaccine against GyH1.

## 2. Materials and Methods

### 2.1. Reagents and Materials

The VP1 coding sequence was codon-optimized without altering the amino acid sequence. Optimization included codon substitution, adjustment of GC content, and removal of certain restriction enzyme recognition sites to enhance gene expression. The optimized GyH1 VP1 gene sequence is provided in Supplementary Table S1. The codon-optimized GyH1 VP1 gene was synthesized by Sangon Biotech Co., Ltd. (Shanghai, China). The cloning vector was pMD-19T Simple Vector (Takara Bio Inc., Kusatsu, Japan), and the expression vector was pSmart I (Sangon Biotech Co., Ltd., Shanghai, China). The VP1 gene was inserted into the pSmart I vector using seamless cloning (Vazyme Biotech Co., Ltd., Nanjing, China) to generate the recombinant expression plasmid pSmart I-VP1, which was confirmed by DNA sequencing. The other materials involved in this study are described in the Supplementary Materials. The *Gyrovirus homsa1* (GyH1) strain LY-GyV3-202201 (GenBank accession no. OQ455056.1) was isolated and maintained by the College of Life Sciences, Longyan University.

### 2.2. Animals

Specific-pathogen-free (SPF) white feather broiler embryos used in this study were purchased from Xinxing Dahuanong Poultry Egg Co., Ltd. (Yunfu, China) and hatched under standard conditions in our laboratory. After one week of acclimation in a controlled isolation environment, healthy 7-day-old chicks with consistent growth status were selected for all experiments. The study protocol (approval no. LY2024031) was reviewed and approved by the Animal Ethics Committee of Longyan University, and all procedures were performed in strict accordance with nationally and internationally recognized guidelines for animal welfare and use.

### 2.3. Expression and Purification of Recombinant VP1 Protein

The recombinant GyH1 VP1 protein was expressed and purified as follows. Using the codon-optimized synthetic VP1 gene as a template, PCR amplification was performed with specific primers carrying homologous arms and *EcoR* V/*Xho* I restriction sites (Table 1). The amplified product was inserted into the pSmart I vector, which had been digested with the corresponding restriction enzymes, via seamless cloning to generate the recombinant expression plasmid pSmart I-VP1. The construct was then verified by DNA sequencing. The correct plasmid was then transformed into *E. coli* BL21 (DE3) competent cells. A single colony was inoculated into LB medium containing kanamycin (50 μg/mL) and cultured at 37 °C until the culture reached an OD600 of 0.6–0.8. Expression was induced by adding IPTG to a final concentration of 0.5 mM for 4 h. The cells were harvested, sonicated, and centrifuged to obtain the protein precipitate, which was inclusion bodies. The inclusion bodies were washed and then dissolved in a binding buffer containing 8 M urea, followed by purification using nickel-nitrilotriacetic acid (Ni-NTA) affinity chromatography. The eluted protein was then subjected to gel filtration chromatography on a Superdex 200 column equilibrated with 50 mM Tris, 300 mM NaCl, 8 M urea (pH 8.0). The peak fractions containing the target protein were collected and pooled. The purified denatured protein was refolded by gradient dialysis sequentially against Tris-HCl buffers (pH 8.5) containing 4 M, 3 M, 2 M, and 1 M urea. The sample was then dialyzed against urea-free buffer overnight, yielding soluble VP1 protein. Protein purity and molecular weight were analyzed by SDS-PAGE. The protein was then specifically identified by Western blotting using a mouse anti-His-Tag monoclonal antibody as the primary antibody and HRP-conjugated goat anti-mouse IgG as the secondary antibody. Protein concentration was determined using a BCA protein assay kit.

### 2.4. Preparation and Characterization of VP1-Loaded Liposomal Nanoparticles (LNP-VP1)

Blank liposomes were prepared using a thin-film dispersion–extrusion method. Hydrogenated soybean phosphatidylcholine (HSPC), sodium 1,2-dimyristoyl-sn-glycero-3-phosphatidylglycerol (DPPG-Na), cholesterol, and egg yolk phosphatidylcholine (Egg PC) were accurately weighed at a molar ratio of 50:25:15:10, with a total lipid-to-planned VP1 protein mass ratio of 10:1. The lipid mixture was dissolved in anhydrous ethanol pre-warmed to 60 °C, and the solution was then shaken in a water bath at the same temperature until complete dissolution was achieved. The solution was then transferred to a round-bottom flask, and rotary evaporation was performed in a 45 °C water bath. First, concentration was carried out under atmospheric pressure at 200 rpm for 5 min, followed by further evaporation under a vacuum of −0.08 MPa for 15–25 min until a uniform lipid film formed on the flask wall. The internal aqueous phase buffer (75 mM sodium citrate, 150 mM citric acid, 5 mM propylene glycol, pH 4.0) pre-warmed to 52 °C was slowly added along the flask wall, and hydration was performed at 52 °C with rotary agitation at 60 rpm for 2 h. The resulting crude suspension was vortexed (1500 rpm, 15 s) and then subjected to probe sonication (200 W, pulsed mode: 5 s on, 25 s off, 4 cycles of 6 pulses each). Finally, the suspension was extruded 15 times through a 0.2 μm polycarbonate membrane at 58 °C to obtain a homogeneous blank liposome suspension.

Subsequently, the external phase of the blank liposomes was exchanged using a Sephadex G-50 gel filtration column. The column was first equilibrated with two column volumes of CAPS buffer (10 mM, pH 9.5). A 1.64 mL aliquot of blank liposomes (the maximum loading volume of the column) was loaded onto the column, and elution was performed with CAPS buffer at a flow rate of approximately 0.25 mL/min. The opalescent fractions exhibiting a clear Tyndall effect were collected, yielding blank liposomes with an internal pH of 4.0 and an external pH of 9.5. The purified VP1 protein was dialyzed against CAPS buffer (pH 9.5) and mixed with the liposome suspension at a protein-to-total-lipid mass ratio of 1:10 (*w*/*w*). The mixture was incubated at 48 °C for 20 min with rotary agitation. This procedure was used as a pH-gradient-assisted protein association/loading approach. Because an intact VP1 protein is unlikely to cross an intact phospholipid bilayer through the conventional ion-trapping mechanism used for small molecules, complete transmembrane transport of VP1 was not assumed. The elevated incubation temperature may increase lipid bilayer fluidity and facilitate the association or partial incorporation of VP1 into the liposomal vesicles.

### 2.5. Purification of the Formulation

To reduce the amount of unassociated VP1 protein, the loading mixture was transferred into a dialysis bag with a molecular weight cutoff of 100 kDa and dialyzed against PBS (pH 7.4) at 4 °C overnight. The material retained within the dialysis bag was collected as the purified LNP-VP1 formulation and stored at 4 °C until further use.

### 2.6. Physicochemical Characterization

Particle size, polydispersity index, and zeta potential: The hydrodynamic diameter, polydispersity index (PDI), and zeta potential of LNP-VP1 were measured at 25 °C using a Malvern Zetasizer. Samples (1 mL) were analyzed without further dilution.

Appearance and colloidal stability observation: Freshly prepared blank liposomes and LNP-VP1 dispersions were visually inspected in transparent glass vials under ambient light. Colloidal formation was qualitatively evaluated by the Tyndall effect using a 650 nm laser beam, with deionized water serving as the negative control.

Morphological observation: Particle morphology was examined by transmission electron microscopy (TEM) after negative staining. Briefly, 10 μL of sample was deposited onto a carbon-coated copper grid and allowed to adsorb for 5 min. Excess liquid was removed, followed by staining with 2% (*w*/*v*) phosphotungstic acid (pH 7.0) for 3 min. The grid was air-dried at room temperature before TEM imaging.

Apparent protein retention efficiency was determined using liposome disruption followed by the BCA assay. The purified LNP-VP1 formulation was disrupted by probe sonication, and the amount of VP1 retained after dialysis was quantified. Apparent protein-retention efficiency was calculated as follows: Apparent protein retention efficiency (%) = (amount of VP1 retained after dialysis/amount of VP1 initially added) × 100%. (This value represents the proportion of VP1 retained with the purified liposomal formulation and may include both internally incorporated and tightly surface-associated protein).

### 2.7. In Vitro Release Study

To evaluate the antigen release behavior of LNP-VP1 under physiological conditions, an in vitro release study was performed in PBS buffer (pH 7.4) at 37 °C. A defined volume of the LNP-VP1 formulation was accurately transferred into a pre-treated dialysis bag (MWCO 100 kDa), sealed after removing air bubbles, and fully immersed in a closed container containing 20 mL of release medium. The container was placed in an air-bath constant temperature shaker at 37 °C with continuous shaking at 110 rpm. Samples were collected intensively at early time points of 0.5, 1, and 2 h, followed by sampling every 2 h thereafter. At each time point, 1.0 mL of the external release medium was withdrawn, and an equal volume of pre-warmed fresh medium was immediately added to maintain a constant total volume and sink conditions. The released VP1 protein concentration in the collected samples was determined using the BCA method. The cumulative release amount (*Q_t_*) and cumulative release rate (*R_t_*) were calculated using the sample replacement formula, and the cumulative release curve was plotted as a function of time. 

Cumulative release amount (*Q_t_*) was calculated using the following formula:

$$Q_{t}\;=\;C_{t}\;\times \;V_{0}+\sum_{i=1}^{n-1}\left(C_{i}\times V_{s}\right)$$

where $Q_{t}$ is the cumulative release amount (μg) at time t, $C_{t}$ is the VP1 concentration (μg/mL) in the external medium at time t, $V_{0}$ is the initial total volume of the release medium (20.0 mL), $C_{i}$ is the VP1 concentration measured at the i-th sampling time point (μg/mL), $V_{s}$ is the sampling volume (1.0 mL) at each time point, and n is the total number of sampling events up to time *t*.

The cumulative release rate (*R_t_*) was calculated using the following formula:

$$R_{t}(\%)\;=\;\frac{Q_{t}}{Q_{\mathrm{total}}}\;\times 100\%$$

where $Q_{t}$ is the cumulative release amount (μg) at time *t*, and $Q_{\mathrm{total}}$ is the total amount of VP1 protein loaded in the LNP-VP1 formulation (μg). All experiments were performed in triplicate, and data were expressed as the mean ± standard deviation (SD).

### 2.8. Preparation of Freund’s Adjuvant-VP1 Vaccine

The purified recombinant VP1 protein solution was mixed with an equal volume of Freund’s complete adjuvant (CFA) for the primary immunization or Freund’s incomplete adjuvant (IFA) for booster immunizations. The mixture was emulsified using an electric homogenizer at 10,000 rpm for 10 min in an ice bath until a stable water-in-oil (W/O) emulsion was formed. The emulsification efficiency was verified by a cold-water stability test, where complete emulsification was confirmed by the absence of droplet diffusion in cold water. Each dose of the prepared vaccine contained 100 μg of VP1 protein in a total volume of 200 μL. The vaccine was stored at 4 °C and used within 24 h.

### 2.9. Grouping and Immunization Schedule

To systematically evaluate the immunogenicity of the LNP-VP1 vaccine, chicks were randomly divided into four groups: PBS negative control group, blank liposome vehicle control group, Freund’s adjuvant-VP1 conventional vaccine group, and LNP-VP1 experimental vaccine group. All groups were immunized via the subcutaneous route. Based on previous studies demonstrating that liposomal nanoparticles as a delivery system significantly enhance the immunogenicity of protein antigens [12], the VP1 antigen dose in the LNP-VP1 group was set to half of that in the conventional Freund’s adjuvant vaccine (50 μg vs. 100 μg), aiming to evaluate its ability to elicit effective immune responses with a reduced antigen dose. The LNP-VP1 group received 50 μg of VP1 per dose, whereas the Freund’s adjuvant-VP1 group received 100 μg per dose. This design was intended as an exploratory antigen-sparing assessment rather than a dose-matched comparison of the two delivery platforms. The detailed design is presented in Table 2. All vaccines or control formulations were administered on day 14 (primary immunization) and day 28 (booster immunization) after hatching.

After the first immunization, the 20 chickens in each treatment group were randomly divided into two predefined cohorts. Ten chickens per group were assigned to the immunological evaluation cohort, and the remaining 10 chickens per group were assigned to the safety evaluation cohort.

The chickens in the immunological evaluation cohort were used for longitudinal blood sampling and for the immunological analyses. These analyses included VP1-specific serum IgG determination, serum cytokine measurement, splenic CD4 and CD8 associated immunofluorescence analysis, and gene expression analysis of the bursa of Fabricius.

The 10 chickens in the safety evaluation cohort were used for terminal gross examination, organ weight measurement, and immune organ index determination. Six chickens were randomly selected from this cohort for histopathological examination. The remaining four chickens were included in the clinical, gross pathological, and organ index evaluations but were not processed for histological examination.

### 2.10. Sample Collection

Serum: Blood was collected from the wing vein before the primary immunization, as well as 7 and 14 days after the primary immunization, and 7 and 14 days after the booster immunization. After standing at room temperature for 1 h, the blood was allowed to clot overnight at 4 °C, and then centrifuged at 3000× *g* for 15 min to separate the serum. The serum was aliquoted and stored at −80 °C.

Peripheral blood: For lymphocyte proliferation assays, anticoagulated blood (using EDTA as an anticoagulant) was collected from the wing vein at the end of the experiment. Blood samples were processed within 2 h after collection for the isolation of peripheral blood mononuclear cells.

Tissue samples: At the end of the experiment, the spleen, thymus, bursa of Fabricius, liver, and kidneys were collected. All tissue samples were immediately immersed in 10% neutral buffered formalin and fixed for 24–48 h. After fixation, part of the spleen tissue was processed for paraffin embedding for immunofluorescence analysis. The remaining tissues (including the rest of the spleen) were subjected to routine paraffin embedding, sectioning, and hematoxylin and eosin (H&E) staining for histopathological examination.

### 2.11. Detection of Serum VP1-Specific IgG Antibodies

Serum VP1-specific IgG antibody: An indirect enzyme-linked immunosorbent assay (ELISA) was performed. A 96-well plate was coated with VP1 protein (4 μg/mL diluted in coating buffer) overnight at 4 °C. After washing, the plate was blocked with 3% non-fat milk for 2 h at room temperature. Diluted serum (The test serum samples were diluted at a ratio of 1:1000 with PBS before being added to the antigen-coated wells) samples were added and incubated for 30 min at 37 °C. Following washing, HRP-conjugated rabbit anti-chicken IgG secondary antibody (diluted 1:5000) was added and incubated for 30 min at 37 °C. Finally, TMB substrate solution was added and incubated for 15 min at room temperature in the dark, and the reaction was stopped with 2 M H_2_SO_4_. The absorbance was read at 450 nm.

### 2.12. Immunofluorescence Analysis of Splenic T Cells

Paraffin-embedded spleen tissues were sectioned into 4 μm thick slices. The sections were deparaffinized, rehydrated, and subjected to antigen retrieval in sodium citrate buffer (pH 6.0), followed by blocking with PBS containing 5% BSA. Mouse anti-chicken CD4 monoclonal antibody and rabbit anti-chicken CD8α monoclonal antibody were used as primary antibodies and incubated with the sections overnight at 4 °C. After washing with PBS, the corresponding fluorescent secondary antibodies Cy3-conjugated goat anti-mouse IgG and Alexa Fluor 488-conjugated goat anti-rabbit IgG were added and incubated for 50 min at room temperature in the dark. Finally, the nuclei were counterstained with DAPI, and the sections were mounted for observation and imaging under a laser scanning confocal microscope. Semi-quantitative analysis of the fluorescence intensity of CD4^+^ and CD8^+^ T cells was performed using ImageJ (version 1.53) software.

### 2.13. Lymphocyte Proliferation Assay

Peripheral blood mononuclear cells (PBMCs) were aseptically isolated from chickens in each group. The cell density was adjusted to 7.5 × 10^6^ cells/mL and seeded into 96-well plates. The cells were stimulated with concanavalin A (ConA, final concentration 8 μg/mL, a T-cell mitogen) or lipopolysaccharide (LPS, final concentration 25 μg/mL, a B-cell mitogen), while unstimulated wells were used as controls. After incubation at 37 °C in a 5% CO_2_ atmosphere for 44 h, 20 μL of CCK-8 solution was added to each well, and the plates were further incubated for 1 h. The absorbance was measured at 450 nm.

### 2.14. Quantification of Serum Cytokines

Commercially available chicken-specific ELISA kits were used to quantify the concentrations of IL-4, IL-6, IL-17, IL2 and IFN-γ in serum at different time points. All assays were performed strictly according to the manufacturer’s instructions.

### 2.15. Quantitative Real-Time PCR Analysis of Gene Expression in the Bursa of Fabricius

Total RNA was extracted from the bursa of Fabricius tissue using the TRIzol method. In brief, the tissue was homogenized in 1 mL of TRIzol reagent. After homogenization, the sample was incubated at room temperature. Then, 0.2 mL of chloroform was added, followed by vigorous shaking for 30 s and incubation at room temperature for 10 min. Then the sample was centrifuged at 12,000× *g* at 4 °C for 15 min to separate the aqueous phase and the organic phase. Then, transfer the upper aqueous phase to a new RNase-free tube, add an equal volume of isopropanol, and incubate at −20 °C for 2 h to precipitate RNA. Then centrifuge and discard the supernatant, wash the RNA pellet with 75% ethanol. Allow the RNA pellet to air-dry slightly and dissolve it in RNase-free water. Measure the concentration for subsequent experiments. Subsequently, the extracted RNA was reverse-transcribed into cDNA and analyzed by real-time quantitative PCR (qPCR). The expression levels of AID, CD40, and IL-10 were normalized to the reference gene. Relative gene expression was calculated using the 2^−ΔΔCt^ method, with the control group used as the calibrator. The primer sequences are listed in Table S2.

### 2.16. Safety Assessment

Throughout the experimental period, the mental status, feed and water intake, fecal characteristics, and injection site reactions (such as redness, swelling, or induration) of the chickens were observed and recorded daily. In addition, individual body weights of all chickens were measured one day before each immunization and daily for three consecutive days after each immunization. Body weight growth curves were plotted and compared among the groups.

At the end of the experiment, after all experimental chickens were euthanized, the thymus, spleen, and bursa of Fabricius were immediately collected. After removing surface blood stains with filter paper, the wet weight of each organ was measured using an electronic analytical balance. The immune organ index was calculated according to the following formula:

$$\mathrm{Immune}\;\mathrm{organ}\;\mathrm{index}\;(g/\mathrm{kg})=\frac{\mathrm{Organ}\;\mathrm{wet}\;\mathrm{weight}\;(g)}{\mathrm{Body}\;\mathrm{weight}\;(\mathrm{kg})}\times 100\%$$

The major immune organs (spleen, thymus, and bursa of Fabricius) and metabolic organs (liver and kidneys), which had been fixed in formalin, were routinely embedded in paraffin and sectioned at a thickness of 4 μm. After deparaffinization and rehydration, the sections were stained with hematoxylin and eosin (H&E). All sections were independently examined by two pathologists blinded to the experimental groups under an optical microscope to evaluate tissue structural integrity, inflammatory cell infiltration, degeneration, necrosis, and other pathological changes. Descriptive analysis and semi-quantitative scoring were performed.

### 2.17. Statistical Analysis

All data were expressed as the mean ± standard error of the mean (SEM). Statistical analyses were performed using GraphPad Prism 9.0 software. Comparisons among multiple groups were conducted using one-way or two-way analysis of variance (ANOVA), followed by Tukey’s or Dunnett’s post hoc test for multiple comparisons. A *p* value of less than 0.05 was considered statistically significant, with significance levels indicated as follows: * *p* < 0.05, ** *p* < 0.01, *** *p* < 0.001, and **** *p* < 0.0001.

## 3. Results

### 3.1. Successful Expression and Purification of Recombinant VP1 Protein

**Figure 1:**
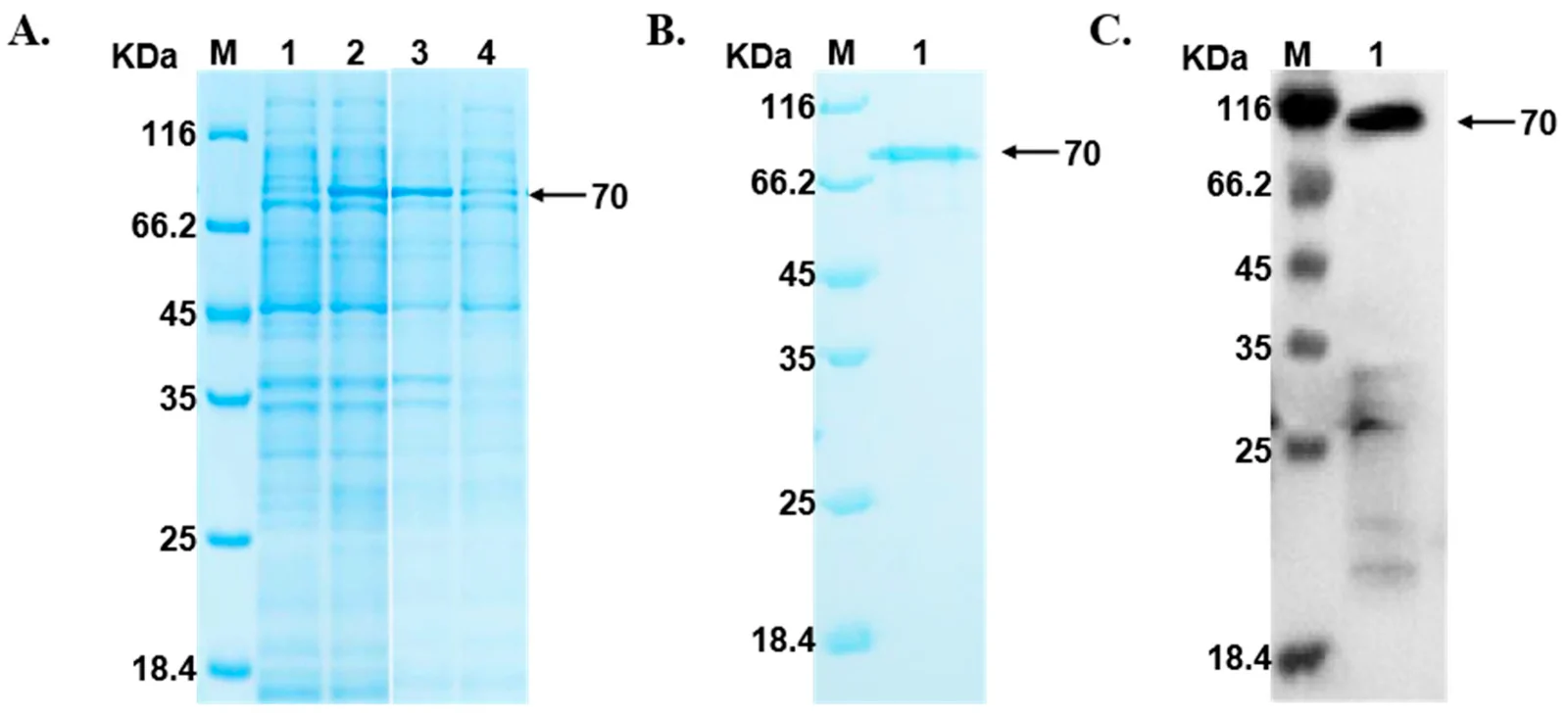
Expression, purification, and immunoblot identification of recombinant GyH1 VP1 protein. (**A**) SDS-PAGE analysis of VP1 protein expression and solubility. M: protein molecular weight marker; 1: uninduced recombinant bacteria (negative control); 2: induced recombinant bacteria (whole-cell lysate, arrow indicates target band); 3: supernatant of cell lysate (soluble fraction); 4: pellet (inclusion bodies). The gel was cropped and spliced from the original gels, and the splicing position is indicated by a thin line. (**B**) SDS-PAGE of the purified and refolded VP1 protein. M: protein molecular weight marker; lane 1: purified and refolded VP1 protein after Ni-NTA affinity and gel filtration chromatography, showing a single band at approximately 70 kDa. (**C**) Western blot validation of the purified VP1 protein. The purified VP1 protein was detected using an anti-His tag monoclonal antibody. M: pre-stained protein molecular weight marker; lanes 1: purified VP1 protein sample, revealing a specific band at approximately 70 kDa.

The recombinant GyH1 VP1 protein was successfully expressed using a prokaryotic expression system, followed by purification and refolding. SDS-PAGE analysis revealed a distinct band at approximately 70 kDa after IPTG induction. This band corresponded to the expected molecular weight of the VP1 protein (including the His-SUMO tag), and the protein was predominantly found in inclusion bodies (Figure 1A). After purification by nickel-affinity chromatography followed by gel filtration chromatography and refolding by gradient dialysis, a highly purified soluble VP1 protein was obtained (Figure 1B). Western blot analysis with an anti-His antibody confirmed the identity of the target protein (Figure 1C). The concentration of the purified protein, determined by using the BCA method, met the requirements for subsequent experiments.

### 3.2. Preparation and Physicochemical Characterization of LNP-VP1

**Figure 2:**
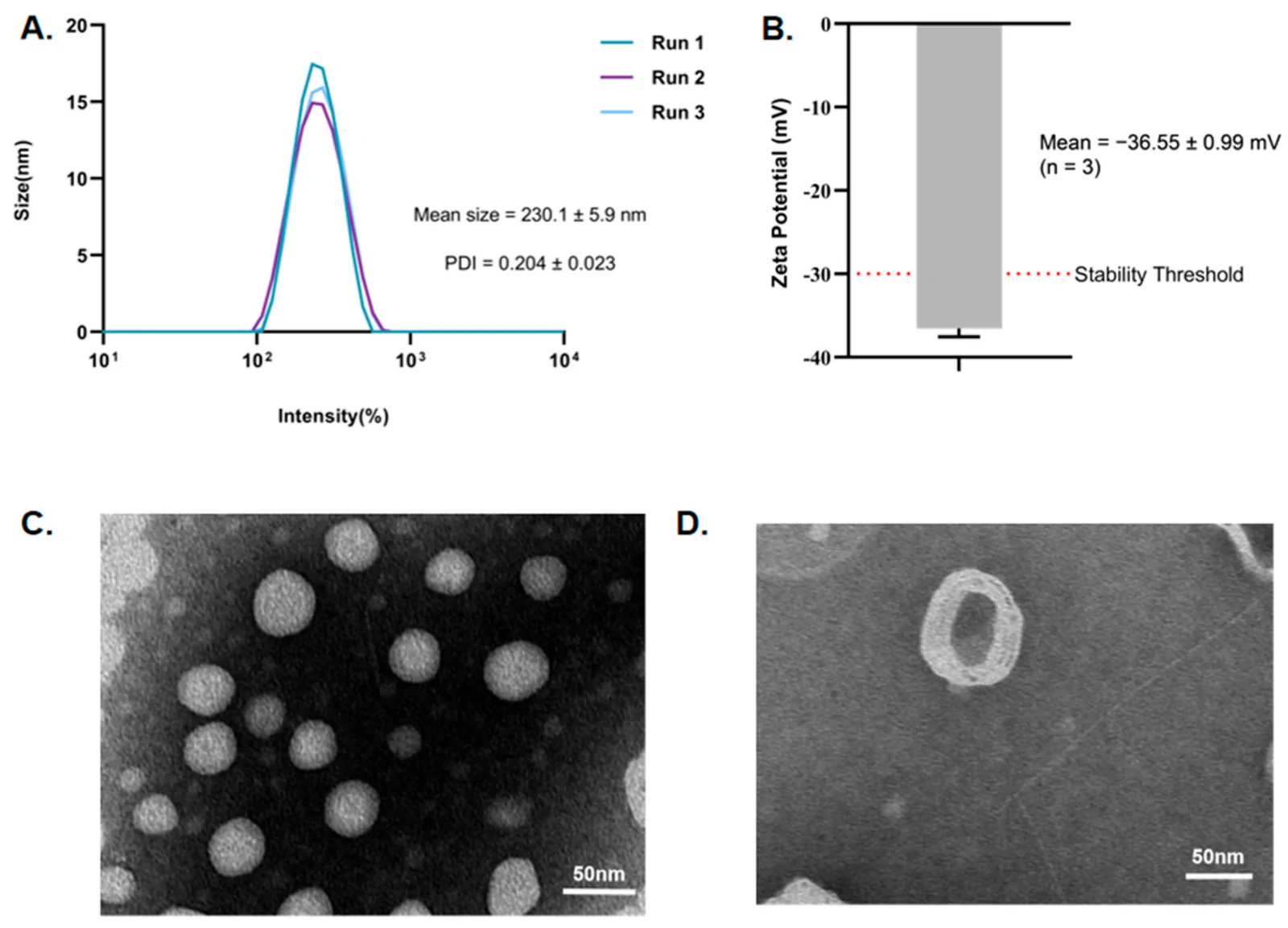
Particle size distribution, zeta potential, and transmission electron microscopy morphology of LNP-VP1. (**A**) Hydrodynamic particle size distribution. (**B**) Zeta potential distribution. Data are presented as mean ± SD (*n* = 3). (**C**,**D**) Representative transmission electron microscopy images of blank liposomes and LNP-VP1. (**C**) Blank liposomes; (**D**) LNP-VP1 Samples were negatively stained with 2% phosphotungstic acid (pH 7.0). Scale bar: 50 nm.

We characterized the physicochemical properties of the LNP-VP1 formulation, including particle size, zeta potential, morphology, and encapsulation efficiency. Dynamic light scattering analysis showed a hydrodynamic diameter of 230.11 ± 5.95 nm and a polydispersity index (PDI) of 0.204 ± 0.023, indicating a homogeneous particle distribution (Figure 2A). The zeta potential was −36.55 ± 0.99 mV, suggesting good colloidal stability (Figure 2B). Transmission electron microscopy revealed that blank liposomes exhibited a typical spherical unilamellar vesicle structure (Figure 2C). LNP-VP1 appeared as spherical vesicles with increased internal electron density after drug loading, which was consistent with the association of VP1 with the liposomal formulation (Figure 2D). The apparent protein-loading efficiency, determined by using the sonication–BCA method, was as high as 84.03% ± 2.1%.

**Figure 3:**
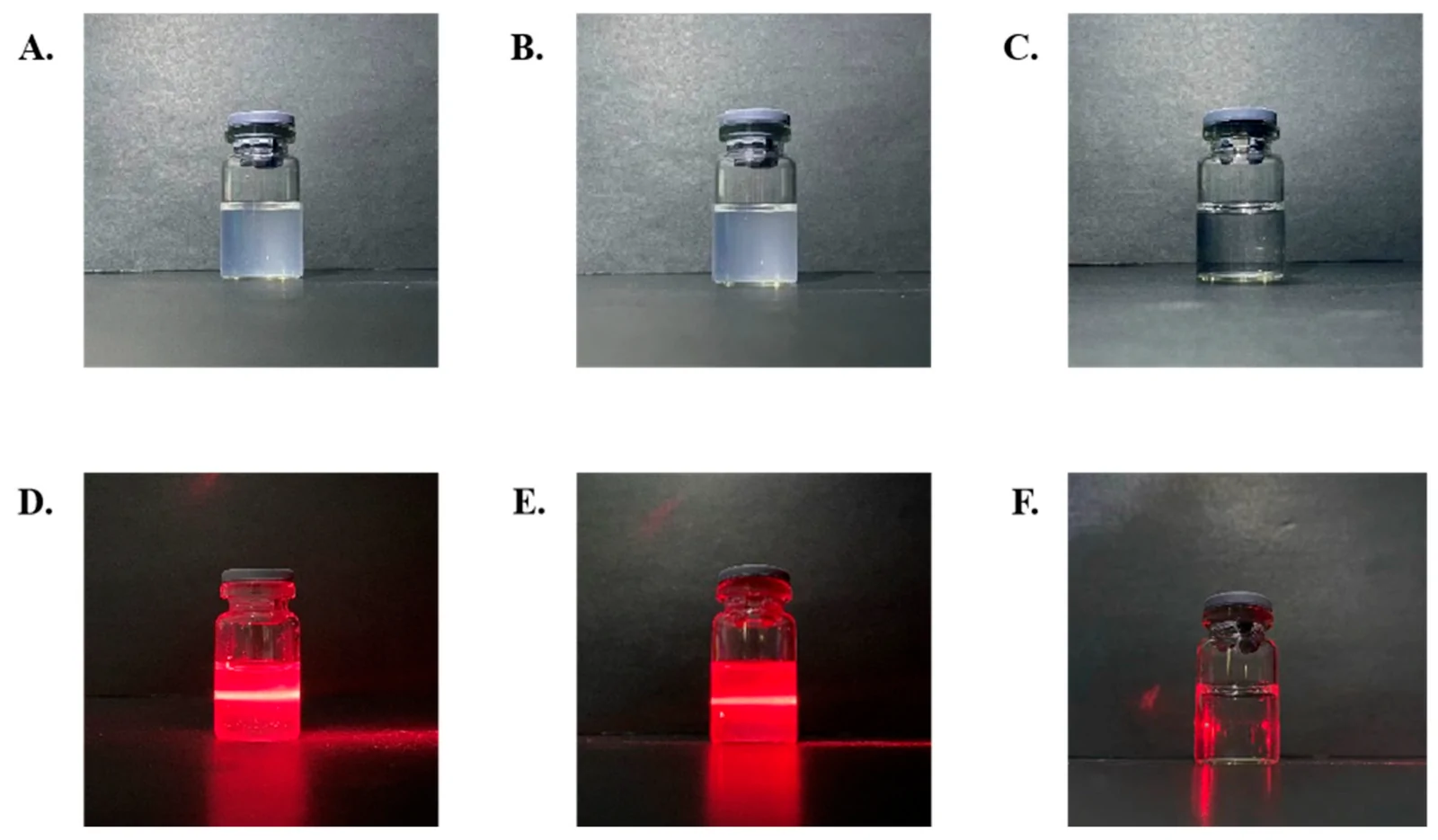
Characterization of liposome appearance and Tyndall effect. (**A**–**C**) Macroscopic appearance under natural light: (**A**) blank liposomes, (**B**) LNP-VP1, and (**C**) water control. Both liposome formulations appeared as milky white, translucent liquids with a faint blue opalescence. (**D**–**F**) Tyndall effect under laser pointer illumination: (**D**) blank liposomes, (**E**) LNP-VP1, and (**F**) water control. The liposome groups exhibited a clear scattered light beam, indicating the formation of stable nano-colloidal dispersion systems; no scattering was observed in the water control.

The macroscopic colloidal properties of the formulations were also evaluated. The appearance and Tyndall effect of blank liposomes (LNP) and VP1-loaded liposomal nanoparticles (LNP-VP1) were observed. As shown in Figure 3A–C, both blank liposomes and LNP-VP1 appeared as milky white, translucent liquids exhibiting a faint blue opalescence under natural light. Upon laser illumination, both formulations exhibited a clear and bright Tyndall scattering beam, whereas the water control showed no scattering (Figure 3D–F). This indicated the formation of stable and homogeneous nano-colloidal dispersion systems.

### 3.3. In Vitro Release Behavior of LNP-VP1

**Figure 4:**
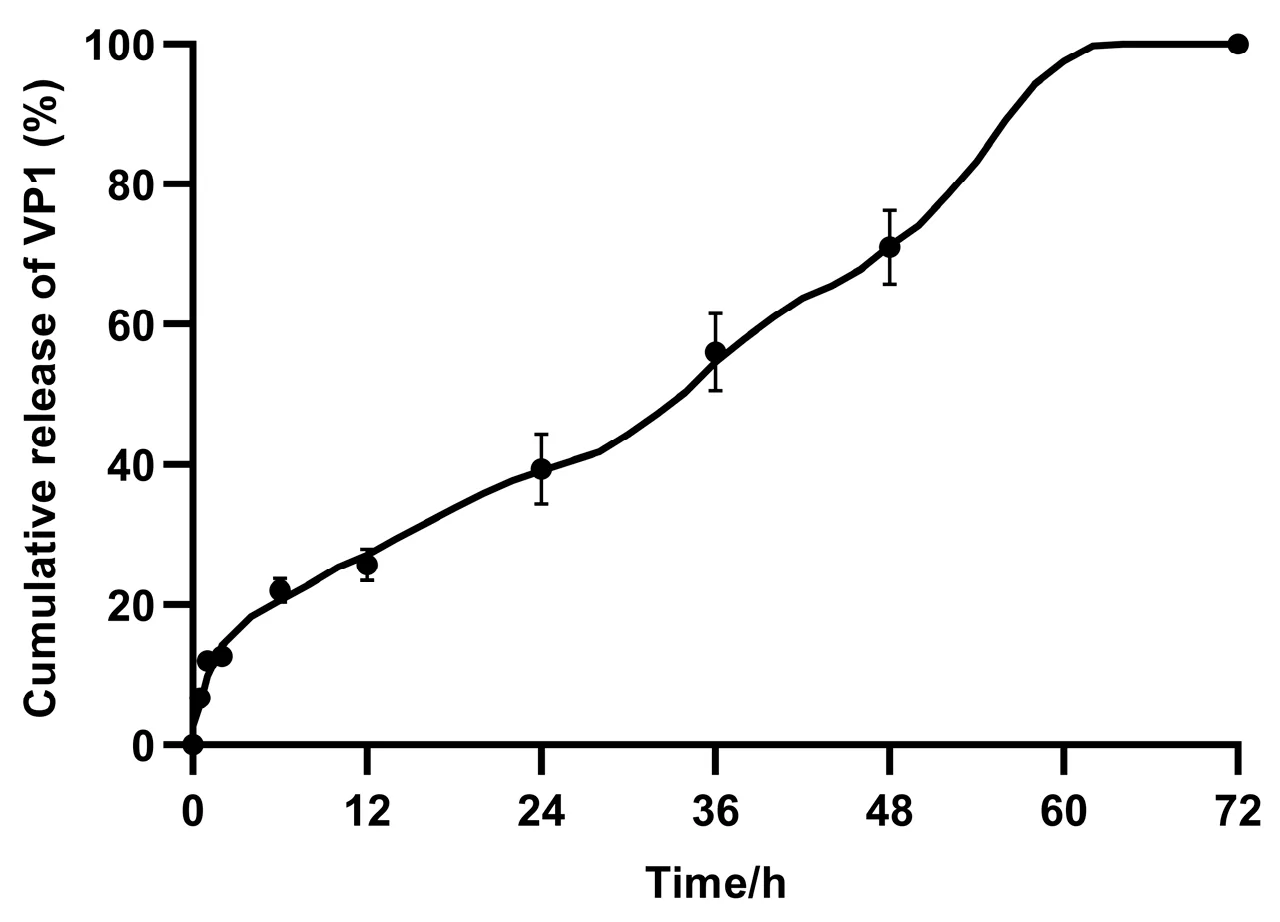
Cumulative in vitro release profile of LNP-VP1 in PBS. *n* = 3; data are presented as mean ± standard deviation (SD).

To evaluate the antigen release profile of LNP-VP1 under physiological conditions, an in vitro release study was conducted in PBS (pH 7.4) at 37 °C. The release profile of LNP-VP1 exhibited an initial burst release followed by a sustained release phase (Figure 4). Approximately 13% of the encapsulated VP1 protein was released within the first 2 h. Subsequently, the formulation entered a prolonged sustained release phase. It achieved a cumulative release rate of over 96% by 58 h, with near-complete release observed at 60 h.

### 3.4. Detection of Humoral Immune Response

**Figure 5:**
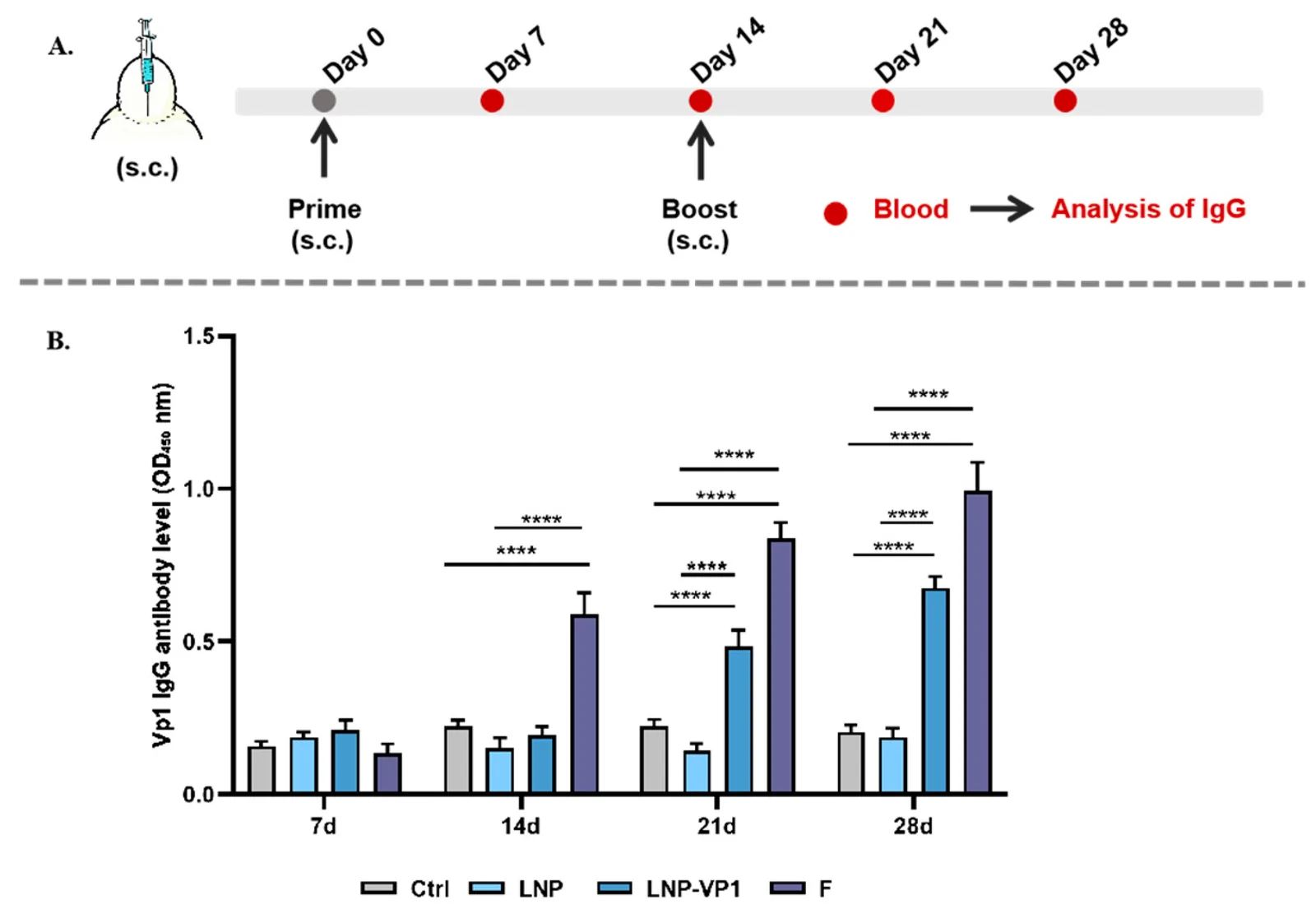
Dynamic changes in VP1-specific IgG antibody titers. (**A**) The immunization schedule and antibody analysis: In the schematic timelines, day 0 represents the day of primary immunization, corresponding to day 14 post hatching. The booster immunization was administered on day 14 after the primary immunization, corresponding to day 28 post hatching. Blood was collected from the wing vein at 7 days and 14 days after the primary immunization, as well as at 7 days and 14 days after the booster immunization. Serum was isolated for the detection of VP1-specific IgG antibodies. (**B**) The serum levels of VP1-specific IgG antibodies in each group. *n* = 10. Data are presented as the mean ± SEM. **** *p* < 0.0001.

To evaluate the level of humoral immune response induced by LNP-VP1, we detected VP1-specific IgG antibodies in serum at different time points after immunization by indirect ELISA. Both the LNP-VP1 vaccine and the conventional Freund’s adjuvant vaccine elicited strong antibody responses (Figure 5). At 14 days after the primary immunization, antibody levels in the Freund’s adjuvant group had already increased significantly. Notably, a marked elevation in antibody titers in the LNP-VP1 group occurred after the booster immunization, and the levels remained stable and high at the end of the experiment. Throughout the monitoring period, antibody titers in the PBS control group and the blank liposome vehicle control group remained at baseline levels with no significant changes.

### 3.5. LNP-VP1 Promotes Cellular Immune Responses

**Figure 6:**
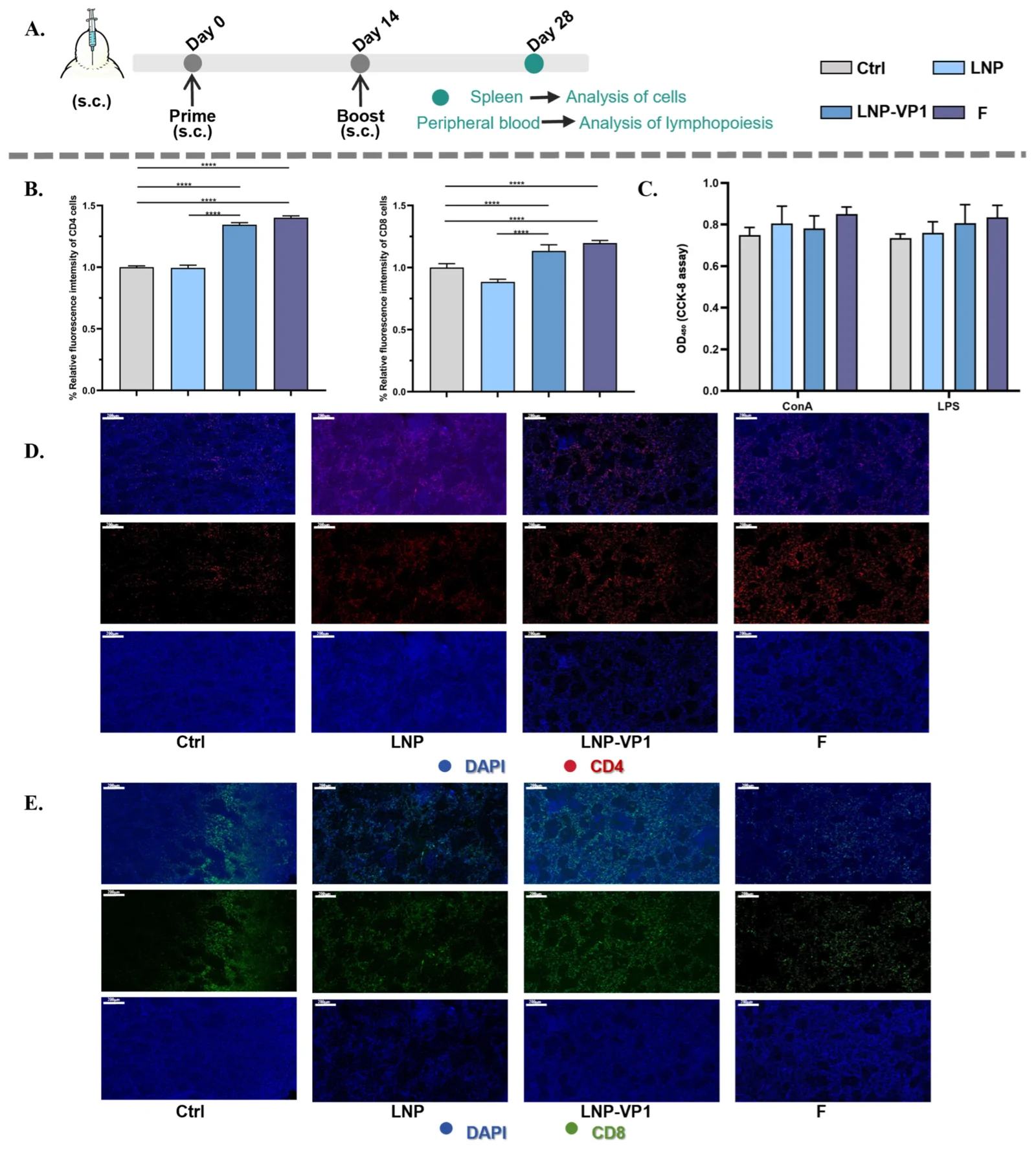
Cellular immune responses induced by LNP-VP1. (**A**) A schematic diagram of the immunization schedule and sampling. (**B**) Semi-quantitative analysis of fluorescence intensity of CD4^+^ and CD8^+^ T cells in the spleen. (**C**) The proliferation levels of peripheral blood lymphocytes. (**D**,**E**) Representative immunofluorescence images of CD4^+^ T cells (**D**) and CD8^+^ T cells in the spleen. Scale bar: 200 μm. *n* = 10. Data are presented as the mean ± SEM. **** *p* < 0.0001.

The distribution and infiltration of CD4^+^ and CD8^+^ T cells in the spleen of each group were examined by immunofluorescence staining. Immunofluorescence analysis (Figure 6B,D,E) showed that, compared with the PBS control group and the LNP alone group, the fluorescence intensities of both CD4^+^ and CD8^+^ T cells in the spleen were significantly enhanced in the LNP-VP1 group and the Freund’s adjuvant group. These results suggest that subcutaneous administration of the LNP-VP1 vaccine effectively induces the activation and expansion of splenic T cells.

To further assess the functional status of vaccine-induced T cells, we evaluated the proliferative capacity of peripheral blood lymphocytes using a CCK-8 assay upon stimulation with ConA or LPS. The results (Figure 6C) showed that the OD_450_ values in the Freund’s adjuvant and LNP-VP1 groups were higher than those in the PBS control group, suggesting a potential immune-enhancing effect.

**Figure 7:**
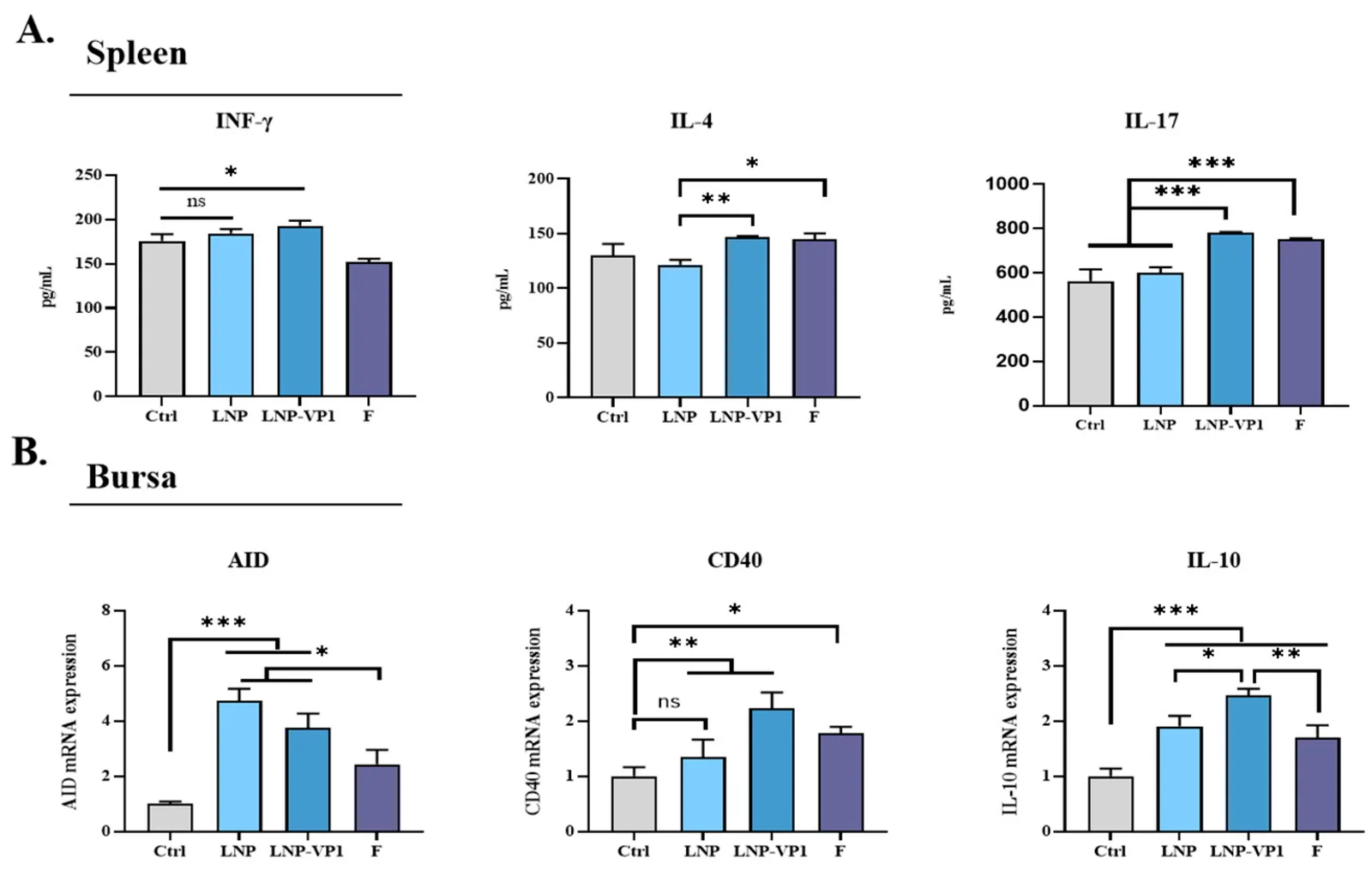
Cytokine Quantification and B-Cell Gene Expression Following LNP-VP1 Immunization. (**A**) Concentrations of key cytokines (IFN-γ, IL-4, IL-17) were measured in serum from Ctrl, Blank LNP, LNP-VP1 and Freund’s adjuvant-VP1 groups. (**B**) mRNA expression levels of AID, CD40, and IL-10 were quantified in bursa of Fabricius. *n* = 10. Data are presented as mean ± SEM. * *p* < 0.05, ** *p* < 0.01, *** *p* < 0.001; ns, not significant.

To further elucidate the polarization of the immune response, the concentrations of key cytokines in serum were quantified. Compared with the Ctrl groups, the LNP-VP1 group exhibited significantly higher serum levels of IFN-γ (*p* < 0.05). In addition, the serum IL-4 and IL-17 levels in the LNP-VP1 group and the Freund’s adjuvant group were significantly higher than those in the blank LNP group (*p* < 0.05) (Figure 7A). The simultaneous elevation of IFN-γ (Th1-type) and IL-4 (Th2-type) suggests that LNP-VP1 successfully induced a robust and balanced cellular and humoral immune response. Notably, the significant increase in IL-17 indicates that the liposomal delivery system may also trigger the Th17 signaling pathway, which is often associated with enhanced mucosal protection and late-stage inflammatory coordination. Moreover, no significant differences were observed in the levels of IL-6 and IL-2 among LNP-VP1 and Ctrl groups (*p* > 0.05) (Supplementary Figure S1). The stable levels of IL-6 suggest that the LNP-VP1 formulation does not induce an over-activated systemic inflammatory storm, while the IL-2 profile might reflect the transient nature of its secretion.

Moreover, as the bursa of Fabricius is the central organ for avian B-cell development, the mRNA expression levels of key regulatory genes were evaluated (Figure 7B). The LNP-VP1 group showed a marked upregulation in the mRNA levels of AID (Activation-induced cytidine deaminase) and CD40 compared to Ctrl groups (*p* < 0.05). AID is a pivotal enzyme for somatic hypermutation and class-switch recombination, while CD40 is essential for T-cell-dependent B-cell activation. Their upregulation suggests that LNP-VP1 promotes high-affinity antibody production and the formation of germinal centers. Interestingly, the expression of IL-10 mRNA was also significantly elevated in the LNP-VP1 group (*p* < 0.05). As a potent anti-inflammatory and regulatory cytokine, the increase in IL-10 in the bursa likely functions as a feedback mechanism to maintain immunological homeostasis and prevent excessive tissue damage during the robust B-cell proliferation phase.

### 3.6. Safety Evaluation

**Figure 8:**
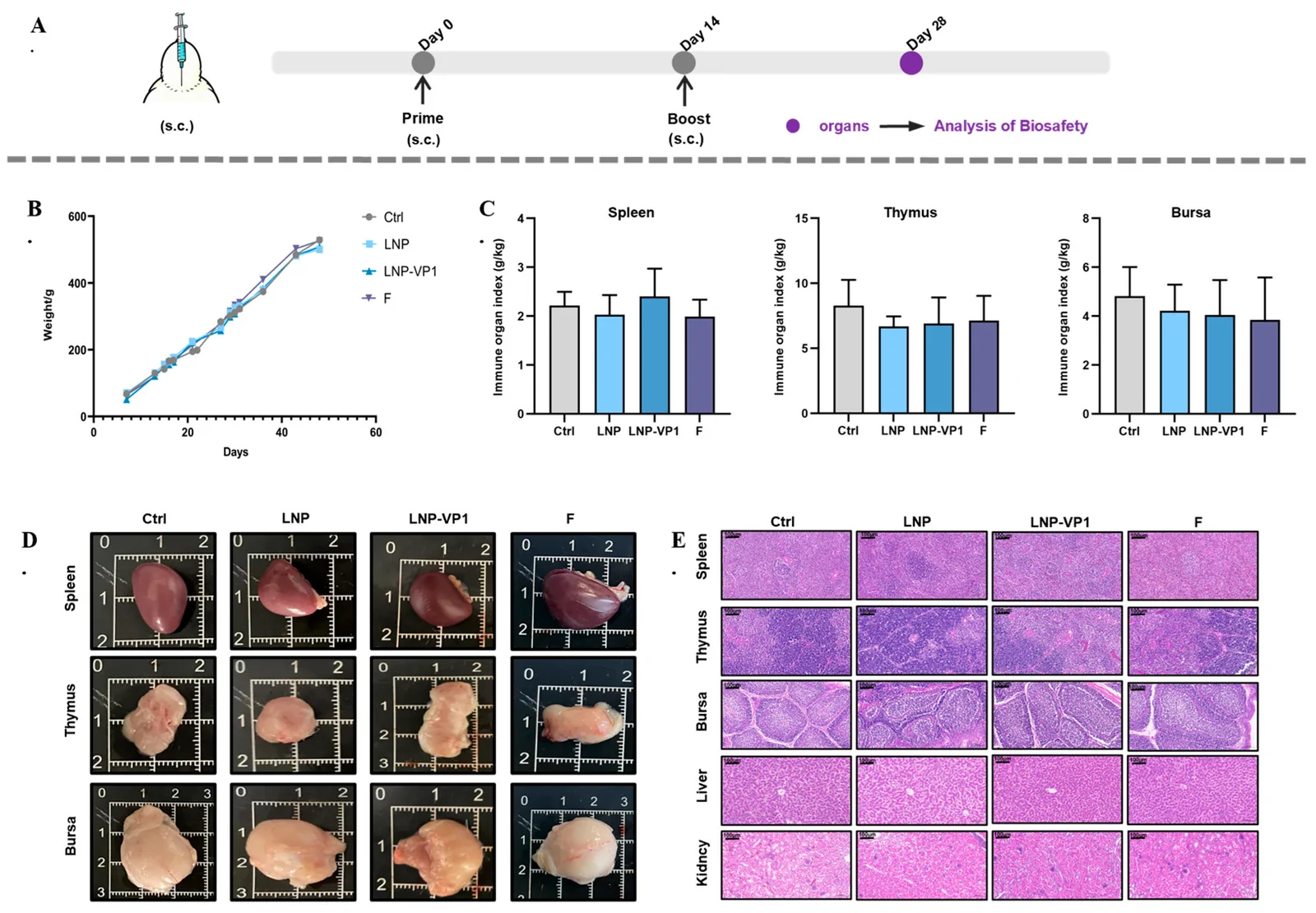
Safety evaluation of LNP-VP1. (**A**) A schematic diagram of the immunization schedule and sampling. (**B**) Body weight growth curves. (**C**) Immune organ indices. The thymus index, spleen index, and bursa of Fabricius index of chickens in each group. (**D**) Macroscopic morphology of major immune organs. (**E**) Histopathological examination of major organs. H&E staining of the spleen, thymus, bursa of Fabricius, liver, and kidneys. Scale bar: 100 μm. *n* = 6. Data are presented as the mean ± SEM.

To assess the in vivo safety of the LNP-VP1 vaccine, we systematically evaluated growth performance, immune organ indices, macroscopic morphology of major immune organs, and histopathology. Growth performance monitoring showed that during the experiment period, the body weight growth curves of chickens in each immunized group were not significantly different from those of the PBS control group (Figure 8B). Immune organ indices revealed that the thymus, spleen, and bursa of Fabricius indices in each group showed no significant differences compared with the PBS control group (Figure 8C). At the experimental endpoint, macroscopic examination of the major immune organs (spleen, thymus, and bursa of Fabricius) showed that organ size, color, texture, and the morphology of both the surface and cut sections in each immunized group were consistent with those of the PBS control group, and no visible abnormalities such as swelling, atrophy, hemorrhage, or nodules were observed (Figure 8D). Histopathological examination demonstrated that in H&E-stained sections of the major immune organs (spleen, thymus, and bursa of Fabricius) and metabolic organs (liver and kidneys), no vaccine-related inflammatory cell infiltration, tissue necrosis, or structural abnormalities were observed in any of the immunized groups (Figure 8E). Together, these results indicate that the LNP-VP1 vaccine has a good safety profile in chickens.

## 4. Discussion

In this study, we systematically evaluated the immunogenicity and safety of a liposomal nanoparticle formulation encapsulating the GyH1 VP1 protein (LNP-VP1) as a novel subunit vaccine in an SPF chicken model for the first time. The prepared LNP-VP1 exhibited a particle size of approximately 230 nm, a polydispersity index as low as 0.204, and a zeta potential of −36.55 mV, characterizing a monodisperse and colloidally stable system. It is well established that nanoscale particles are more efficiently taken up by antigen-presenting cells and drained to lymphoid tissues, thereby enhancing vaccine immunogenicity [13]. Our formulation demonstrated a characteristic initial burst release followed by a zero-order sustained release kinetic, achieving near-complete cumulative release (>96%) within 58 h. This release profile likely establishes a “depot effect” in vivo, ensuring prolonged antigen exposure. Such sustained delivery is known to optimize germinal center (GC) reactions, which are indispensable for the induction of high-affinity, long-lasting antibody responses [14]. Moreover, LNP-VP1 exhibited a clear Tyndall effect under laser illumination, confirming the formation of a stable nano-colloidal dispersion system. Collectively, these physicochemical properties provide a solid foundation for the immune delivery function of LNP-VP1.

Humoral immunity is a core indicator for evaluating vaccine protective efficacy, and the level of specific IgG antibodies directly reflects the extent of B-cell activation and the formation of immunological memory [15,16]. Our dynamic monitoring revealed distinct kinetic profiles between the LNP-VP1 and Freund’s adjuvant groups. While Freund’s adjuvant elicited a rapid increase in antibodies by day 14 post priming, the LNP-VP1 group exhibited a more gradual initial rise followed by a robust and stable surge post booster. This “delayed-onset, high-persistence” profile aligns with the sustained release behavior observed in vitro. Although LNPs primarily facilitate antigen presentation within draining lymph nodes, their ability to prolong the availability of the antigen optimizes the maturation of long-lived plasma cells and memory B cells [7,17]. Notably, the 50 μg LNP-VP1 formulation elicited sustained VP1-specific IgG responses, whereas the Freund’s adjuvant formulation contained 100 μg of VP1. This finding suggests that LNP-VP1 remained immunogenic at the lower dose examined. However, because the two formulations contained different antigen doses, antigen dose and delivery platform were confounded in the present experimental design. Therefore, these results cannot establish an antigen-sparing effect or demonstrate the equivalence or superiority of the LNP formulation relative to Freund’s adjuvant. Future studies should include dose-matched groups and formal dose–response comparisons using multiple VP1 doses with each delivery platform.

Beyond humoral responses, cellular immunity is critical for the clearance of viral reservoirs and the establishment of comprehensive protection [18]. The spleen, as the largest secondary lymphoid organ, serves as a central hub for the activation and proliferation of T and B cells [19,20]. An ideal adjuvant should effectively prime both CD4^+^ helper T cells and CD8^+^ cytotoxic T cells [21], which play essential roles in coordinating adaptive immune responses and eliminating viral infections [22]. In the current study, LNP-VP1 significantly increased the infiltration and fluorescence intensity of CD4^+^ and CD8^+^ T cells in the spleen, matching the performance of the traditional Freund’s adjuvant. While the proliferative capacity of peripheral blood lymphocytes showed only a trending increase without reaching statistical significance, this likely reflects the temporal and spatial distribution of the immune response. Given that peripheral blood contains only a minute fraction (~2%) of the total lymphocyte pool [23,24], the marked T-cell enrichment in the spleen provides the most compelling evidence of a potent cellular immune response.

Upon antigen stimulation, CD4^+^ T cells can differentiate into distinct subsets, including Th1 and Th2 cells, and exert immunoregulatory functions through the secretion of signature cytokines [25,26]. To further elucidate the T-cell polarization induced by LNP-VP1, we analyzed the signature cytokines of the Th1, Th2, and Th17 lineages. The concurrent elevation of IFN-γ (Th1) and IL-4 (Th2) indicates that LNP-VP1 triggers a balanced, multifaceted adaptive response. Crucially, the vaccine elicited a robust IL-17 response. As a hallmark of Th17-mediated immunity, IL-17 is pivotal for mucosal defense, driving neutrophil recruitment and reinforcing epithelial barrier integrity [27,28]. Although GyH1 is primarily a gastrointestinal pathogen, our results demonstrate that subcutaneous LNP-VP1 administration can prime a systemic Th17 response, suggesting potential cross-protection at mucosal surfaces. This finding opens a promising avenue for exploring mucosal delivery routes to further enhance local immunity against mucosal-tropic pathogens.

The molecular analysis of the bursa of Fabricius provides deeper insights into the quality of the induced B-cell response. The significant upregulation of AID mRNA—a key enzyme in somatic hypermutation and class-switch recombination—indicates that LNP-VP1 facilitates antibody affinity maturation. This implies that the resulting antibodies may possess superior neutralizing capacity. Furthermore, the rise in CD40 expression confirms efficient T-B-dcell cross-talk within the bursal microenvironment. Importantly, the elevation of IL-10 suggests a sophisticated homeostatic mechanism; by modulating pro-inflammatory signals (such as IFN-γ or IL-17), IL-10 ensures a stable environment for B-cell differentiation, preventing the immunopathology often associated with potent adjuvants. This is supported by the stability of IL-6 levels, which suggests that the formulation avoids triggering a systemic “cytokine storm” or acute inflammatory distress [29].

In terms of safety assessment, LNP-VP1 exhibited an excellent biocompatibility profile. LNP-VP1 did not adversely affect the growth performance of chickens throughout the experimental period. Immune organ indices showed no significant differences compared with the control group, and no vaccine-related macroscopic or histopathological changes were observed in the major immune organs (spleen, thymus, and bursa of Fabricius) or metabolic organs (liver and kidneys). This safety profile is particularly important for poultry vaccines, as excessive inflammatory responses not only compromise animal welfare but may also lead to reduced production performance.

## 5. Conclusions

In this study, we developed a liposomal formulation containing the GyH1 VP1 protein (LNP-VP1). The formulation exhibited favorable physicochemical stability and sustained release characteristics. In the SPF chicken model, LNP-VP1 induced VP1-specific IgG antibody responses and enhanced splenic infiltration of CD4^+^ and CD8^+^ T cells. Cytokine profiling showed that LNP-VP1 could significantly induce Th1 and Th2-type responses. Notably, it elicited a robust IL-17 response. Safety assessment showed no elevation of IL-6 levels, no significant changes in immune organ indices, and no vaccine-related histopathological alterations in major organs. Collectively, this study provides a candidate vaccine for GyH1 control and experimental evidence for liposomal nanoparticles as a delivery platform for avian subunit vaccines. However, protective efficacy has not yet been established. Future studies should include virus-neutralization assays and GyH1 challenge experiments to evaluate protection against infection and disease.

## Figures and Tables

**Table 1:** Primer sequences used for cloning of GyH1 VP1 gene.

Primer Name	Sequence (5′→3′)	Amplicon Size (bp)
GyH1-VP1-F	TCGAGCTCCGTCGACGATATCGCGCGTCGTTTCCGCCGC	1300
GyH1-VP1-R	GTGGTGGTGGTGGTGCTCGAGTTCCAGATCAGCCGGAACAC	

Notes: Underlined sequences indicate restriction enzyme sites for directional cloning. The sequences preceding the sites are homologous arms for seamless cloning.

**Table 2:** Experimental design and immunization schedule in chickens.

Group	*n*	Vaccine Content	VP1 Dose	Route	Immunization Schedule (Day Post Hatching)
Control	20	PBS	0 µg	SC	14 (Prime), 28 (Boost)
Empty LNP (Carrier)	20	Blank LNP	0 µg	SC	14 (Prime), 28 (Boost)
Conventional VP1 Vaccine	20	VP1 + Freund’s Adjuvant	100 µg	SC	14 (Prime), 28 (Boost)
LNP-VP1	20	LNP-encapsulated VP1	50 µg	SC	14 (Prime), 28 (Boost)

Note: SC, subcutaneous injection; *n*, number of animals per group; Prime, primary immunization; Boost, booster immunization.

## Data Availability

The original contributions presented in this study are included in the article. Further inquiries can be directed to the corresponding author.

## Supplementary Materials

The following supporting information can be downloaded at: https://www.mdpi.com/article/10.3390/vetsci13080801/s1, Figure S1: Serum cytokine levels. Serum cytokine levels were measured by ELISA. (A) IL-2, (B) IL-6. Data are presented as mean ± SEM. n = 6; Table S1: Codon-optimized nucleotide sequence of the GyH1 VP1 gene; Table S2: Primer sequences used for quantitative real-time PCR.

## Abbreviations

GyH1, *Gyrovirus homsa1*; VP1, capsid protein; LNP, liposomal nanoparticle; CFA, complete Freund’s adjuvant; IFN-γ, interferon-gamma; IL-2, interleukin-2; IL-4, interleukin-4; IL-6, interleukin-6; IL-17, interleukin-17; Th1, T helper type 1; Th2, T helper type 2; Th17, T helper type 17; H&E, hematoxylin and eosin; CAPS, 3-(cyclohexylamino)-1-propanesulfonic acid; HSPC, hydrogenated soybean phosphatidylcholine; DPPG-Na, sodium 1,2-dimyristoyl-sn-glycero-3-phosphatidylglycerol; Egg PC, egg yolk phosphatidylcholine.
