# Supplementary material for: Enhanced Humoral and Cellular Immune Responses Elicited by a Liposomal Subunit Vaccine Based on Gyrovirus homsa1 VP1 Protein in Chickens
Source: Vet Sci. 2026 Aug 13;13(8):801. doi: 10.3390/vetsci13080801 (PMC13517395; doi:10.3390/vetsci13080801)
Supplement: Supplementary file 1 [file vetsci-13-00801-s001.zip › vetsci-4437072-supplementary.pdf]

Supplementary information

Enhanced Humoral and Cellular Immune Responses Elicited by A Liposomal Subunit Vaccine Based on *Gyrovirus homsa1* VP1 Protein in Chickens

Mengshi Chen[1,#], Rongchang Liu[2,#], Xin Yang[1,#], Chuchu Duan[1,3,#], Xiaozhen Yu[1], Liyun Zhuang[1,3], Tingting Dai[1,3], Haiyu Chen[1,3], Zehua Jin[1], Zucheng Song[1,*], Xintian Zheng[1,*]

1 Fujian Provincial Key Laboratory of Preventive Veterinary Medicine and Veterinary Biotechnology, College of Life Sciences, Longyan University, Longyan 364000, China

2 Fujian Provincial Key Laboratory for Avian Diseases Control and Prevention, Institute of Animal Husbandry and Veterinary Medicine of Fujian Academy of Agricultural Sciences, Fuzhou, 350000, China

3 College of Animal Sciences, Fujian Agriculture and Forestry University, Fuzhou 350000, China

# These authors contributed equally to this work.

* Correspondence and reprint requests: Xintian Zheng: College of Life Sciences, Longyan University, Longyan 364000, Fujian, P.R. China; xintianzheng@lyun.edu.cn. Zucheng Song: College of Life Sciences, Longyan University, Longyan 364000, Fujian, P.R. China; s1271967668@163.com

Materials and methods

Materials

The primary reagents and materials used in this study were as follows. For molecular cloning and protein expression, a seamless cloning kit (Vazyme Biotech Co., Ltd., Nanjing, China), restriction endonucleases EcoR V and Xho I (Takara Bio Inc., Kusatsu, Japan), isopropyl-β-D-thiogalactopyranoside (IPTG, ≥99%, Sangon Biotech Co., Ltd., Shanghai, China), lysozyme (Sangon Biotech Co., Ltd., Shanghai, China), a Ni-NTA protein purification kit (BBI Life Sciences Corp., Shanghai, China), and a BCA protein assay kit (Fujian Herui Biotechnology Co., Ltd., Fujian, China) were used. A Sephadex G-50 gel filtration column (particle size 50–150 μm, separation range 1500–3×10^4, bed volume 8.3 mL, Wuxi Tianyan Biotechnology Co., Ltd., Wuxi, China) was used for desalting and molecular sieving. For liposome preparation, hydrogenated soybean phosphatidylcholine (HSPC, Shanghai Macklin Biochemical Co., Ltd., Shanghai, China), sodium 1,2-dimyristoyl-sn-glycero-3-phosphatidylglycerol (DPPG-Na, ≥99%, Shanghai Aladdin Biochemical Technology Co., Ltd., Shanghai, China), cholesterol (Sangon Biotech Co., Ltd., Shanghai, China), egg yolk phosphatidylcholine (Egg PC, Beijing Solarbio Science & Technology Co., Ltd., Beijing, China), 3-(cyclohexylamino)-1-propanesulfonic acid (CAPS, Shanghai Aladdin Biochemical Technology Co., Ltd., Shanghai, China), and Triton X-100 (Beijing Solarbio Science & Technology Co., Ltd., Beijing, China) were used. For animal immunization and immunological assays, complete Freund's adjuvant and incomplete Freund's adjuvant

(Sigma-Aldrich, St. Louis, MO, USA), horseradish peroxidase (HRP)-conjugated rabbit anti-chicken IgG antibody (BBI Life Sciences Corp., Shanghai, China), mouse anti-chicken CD4 monoclonal antibody (Abcam, Cambridge, UK), rabbit anti-chicken CD8α monoclonal antibody (Abcam, Cambridge, UK), DAPI staining solution and anti-fade mounting medium (Wuhan Boerfu Biotechnology Co., Ltd., Wuhan, China), lipopolysaccharide (LPS, Beijing Solarbio Science & Technology Co., Ltd., Beijing, China), human peripheral blood lymphocyte separation medium (TBD Science, Tianjin, China), concanavalin A IV (ConA, Beijing Solarbio Science & Technology Co., Ltd., Beijing, China), RPMI 1640 medium and enhanced cell counting kit-8 (CCK-8, Dalian Meilun Biotechnology Co., Ltd., Dalian, China), chicken specific cytokine (IL-4, IL-6, IL-17, IFN-γ) ELISA kits (Shanghai Enzyme-linked Biotechnology Co., Ltd., Shanghai, China), 10 × ELISA coating solution, TMB substrate, nitrocellulose membrane and polyvinylidene difluoride (PVDF) membrane (Merck Millipore, Burlington, MA, USA), as well as routine reagents for SDS-PAGE, western blotting, and ELISA were purchased from Beijing Solarbio Science & Technology Co., Ltd. (Beijing, China).

Table S1. Codon-optimized nucleotide sequence of the GyH1 VP1 gene

Gene	Optimized Codon
VP1	GCGCGTCGTTTCCGCCGCCACCGTGGTAAATTCGGGTACTATCGTCGTGGCCGTTG
	GCACTGGCGCCACCGTCTGCGTCGTCGTCGTTATAGCCGCCGTCGTAAACTGCGTT
	ACGCGCGTCGTCGTCCGTCTGCACGTAGCGTTAAACGTAAAATCTTCAACCCGCAT
	CCGGGTAGTTACCTGGTTCGTCTGCCGAACCCGTACAACGCTATCAACCTGTACTTC
	CAGGGTCTGGTGTTCATCCCGCGTGCGACCTCTTACCTGCCGGATACCACCAAAGG
	CAAAAACGTTACCACCACCAACGTTGCGCTGATCAACGTTAACCTGAAAGAGTTCT
	TCTGGGCGACCCTGCCGCTGGATGCGCGCTCTAAAATCGGCGGTCCGAACCCGTTC
	CCGCAGCACATCCAGGGCTGCGATTGGGCGGGCATCGCGACCACCCACAAAGGCA
	CCTGGCCGTACTCTACCCAGATGAGCAGCGCTCGTCAGCCGGGCGCTTGGTCTAGC
	GAATGGTGGCGTTGGGCGCTGCTGCTGATGCACCCGCGTTCTAACGTTCGTTTCTT
	CGGCTCTCCGAAACTGATGACCCTGTCTCAGATCGGCCAGTTCCTGGGTGGTTGGC
	AGCTGTTCACCCACCGTTTCACCAAATTCCGCGTGCTGGCGACCAAATCTCGTGAA
	TCCTTCAGCCCGGTTGCTTCTCTGCTGGTTCAGGACAACTACTTCGCGCGTCGCGA
	AGGTGCAGGCCCGCCGATCTCTGGTCAGCCGCCGATGTGCACCATGCAGCGTCTGA
	CCCGTGATTATTCTGGCACCGAATCCAACGCGCCGGCGAACGAAACCACCATCCCG
	TCCATGCCGCCGGACCCGCCGCAGTACCCGGTGCAGACCGGTTGCAGCACTAGCG
	CGGACCCAGGTGAATACCTGCTGGCGGGCCTGACCCGTACCGCGGTTAGCTGCTGG
	TACAGCCGTTCTACCTATCCGAGCCTGGCTACTCTGTCTGCGCTGGGCGCTCCGTGG
	TCCTTCCCGGCGGGTCAGAAAAGCATCTCCAAAACCAGCTTCAACAAACACGTGA
	TCCGTGGCATGGGTGATCCGCAGGGTAAAAAATGGCTGACCCTGGTTCCGAAAGG
	TCAGGAATGGATCAACGCTGATTCTATGACTAAAAGCGAACTGGATACCGACATCG
	CCACCCTGTACCTGGCGCAGGGCACCTCCCGTGCTAACTCCTGTAGACAAATTCAA
	CACCTTCCACGAAGTTATGGTCCAGGACCCGATGAACGTTGCACCGTGGGCGGTTG
	TTAAAGTTAGCAGCGTGTGGACCCTGGGCAACAACCGTCGCCCGTACCCGTGGGA
	CGTTAACTGGTATAACGAATTTACCGCGGAAGGTCGTGTTCCGGCTGATCTGGAA

Table S2. Primer sequences used for quantitative real-time PCR

Gene	Sequence (5′–3′)
AID-forward	GCGTAACAAGATGGGTTGCC
AID-reverse	GGGTAGGCACGAAGGAAGTC
CD40-forward	GGGCTCGTGGTGAAGGTGAAAG
CD40-reverse	GGATCAGCACTGACAGCGATGAG
IL10-forward	CGCTGTCACCGCTTCTTCA
IL10-reverse	TCCCGTTCTCATCCATCTTCTC
β-actin-forward	GAGAATTGTGCGTGACATCA
β-actin-reverse	CCTGAACCTCTCATTGCCA

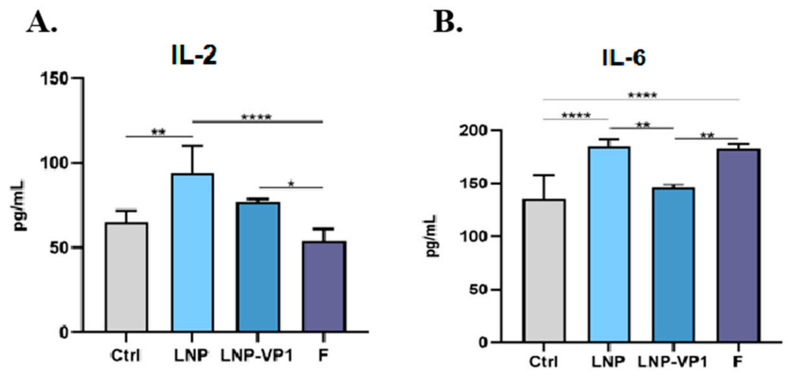

Figure S1. Serum cytokine levels. Serum cytokine levels were measured by ELISA. (A) IL-2, (B) IL-6. Data are presented as mean ± SEM. n=6.
